# Grafting Dendrons onto Pillar[5]Arene Scaffolds †

**DOI:** 10.3390/molecules26082358

**Published:** 2021-04-18

**Authors:** Iwona Nierengarten, Michel Holler, Marine Rémy, Uwe Hahn, Aurélien Billot, Robert Deschenaux, Jean-François Nierengarten

**Affiliations:** 1 Laboratoire de Chimie des Matériaux Moléculaires, Université de Strasbourg et CNRS (UMR 7402 LIMA), Ecole Européenne de Chimie, Polymères et Matériaux, 25 rue Becquerel, CEDEX 2, 67087 Strasbourg, France; iosinska@unistra.fr (I.N.); mholler@unistra.fr (M.H.); marine.remy@etu.unistra.fr (M.R.); u.hahn@unistra.fr (U.H.); 2 Institut de Chimie, Université de Neuchâtel, Avenue de Bellevaux 51, 2000 Neuchâtel, Switzerland; aurelien.billot@unine.ch

**Keywords:** pillar[5]arene, dendrimers, liquid crystals, transfection, glycoclusters

## Abstract

With their ten peripheral substituents, pillar[5]arenes are attractive compact scaffolds for the construction of nanomaterials with a controlled number of functional groups distributed around the macrocyclic core. This review paper is focused on the functionalization of pillar[5]arene derivatives with small dendrons to generate dendrimer-like nanomaterials and bioactive compounds. Examples include non-viral gene vectors, bioactive glycoclusters, and liquid-crystalline materials.

## 1. Introduction

Dendrimers have been intensively investigated during the past decades and this field largely crossed the boundaries between chemistry and other disciplines such as physics and biology [1,2,3,4,5]. This paper being part of the special issue dedicated to Jean-Pierre Majoral, it must be mentioned that his group played a major role in dendrimer chemistry with the development of phosphorus dendrimers [6]. This beautiful chemistry has been used for the construction of a very large variety of functional nanomaterials and bioactive molecules [7,8,9,10,11,12,13,14]. As far as the synthesis of dendrimers is concerned, the convergent and divergent stepwise approaches used for their preparation are often efficient but the synthesis of high generation derivatives remains often difficult because of the large number of synthetic steps [1,2,3,4,5]. An alternative approach to construct large molecules in a rapid manner emerged in recent years and is based on the grafting of small dendrons to compact multifunctional molecular scaffolds to generate globular dendrimer-like compounds in a single step [15,16,17,18,19,20,21,22,23,24,25,26]. As part of this research, our group has intensively investigated fullerene hexa-adducts for this purpose [15,16,17,18]. The grafting of twelve peripheral groups onto the fullerene scaffold has been efficiently achieved by using click chemistry, thus giving rapid access to globular multifunctional nanomaterials [26,27,28,29,30,31,32,33,34,35]. Easy accessibility has been a clear advantage for their applicability in various fields. Examples include solar energy concentrators to improve the efficiency of photovoltaic cells [36], liquid-crystalline materials [37,38], bioactive glycoclusters [39,40,41,42,43,44,45], giant molecules with antiviral properties [46,47], and non-viral gene transfection vectors [48]. More recently, we have also shown that clickable pillar[5]arene scaffolds are versatile compact cores for the preparation of dendrimer-like compounds for various applications. These results are summarized in the present paper.

## 2. Clickable Pillar[5]Arene Building Blocks

**Figure 1:**
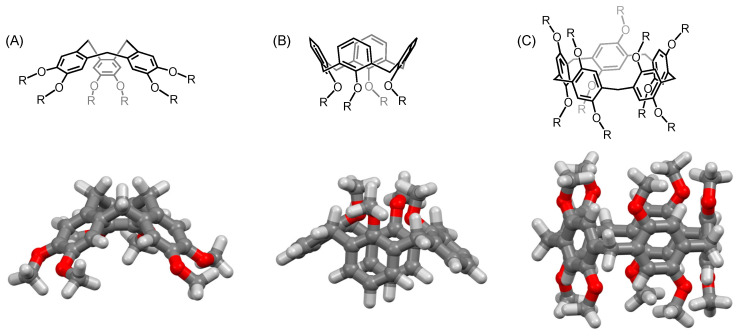
(**A**) Cyclotriveratrylene; (**B**) calix[4]arene; (**C**) pillar[5]arene. The calculated structures of the methoxy-substituted derivatives highlight the different conformations adopted by these macrocycles: cone-shape in the case of the CTV and the calix[4]arene, and tubular in the case of the pillar[5]arene.

Pillar[5]arenes are macrocyclic compounds composed of hydroquinone units connected by methylene bridges [49,50,51,52] (Figure 1). In this respect, they are directly related to cyclotriveratrylenes (CTVs) and calix[*n*]arenes. These three classes of cyclophanes differ by the relative position of the methylene moieties bridging their aromatic subunits. Their shape is also very different. Whereas CTVs and calix[*n*]arenes generally adopt cone-shaped conformations, pillar[5]arenes are tubular-shaped [50,51,52]. As a result, both rims of the pillar[5]arenes are equivalent. With their ten peripheral alkoxy substituents, these compounds are therefore attractive compact scaffolds for the construction of nanomaterials with a controlled number of functional groups distributed around the macrocyclic core.

**Figure 2:**
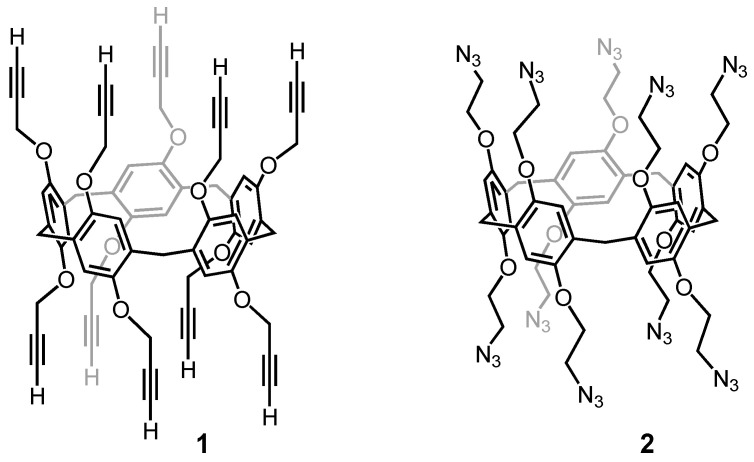
Typical examples of clickable pillar[5]arene derivatives used for the construction of multifunctional nanomaterials.

As far as their synthesis is concerned, pillar[5]arenes are conveniently prepared from 1,4-dialkoxybenzene and paraformaldehyde in the presence of a catalyst, typically BF_3_.Et_2_O [53]. The outcome of the reaction is sensitive to the solvent and CH_2_Cl_2_ or 1,2-dichloroethane favors the formation of the cyclopentamers [54]. In contrast, cyclohexameric macrocycles are preferentially formed in CHCl_3_ [55]. It is believed that the solvent molecules are somehow templating the cyclization step to preferentially generate a macrocyclic product with the appropriate size for inclusion of a solvent molecule in its cavity [54]. On the other hand, the yield of these cyclooligomerizations is particularly high owing to the reversibility of the Friedel–Crafts reaction [56]. The formation of pillar[5]arenes is indeed thermodynamically driven and their high yielding synthesis is explained by dynamic covalent bond formation [56]. Their preparation is, however, very sensitive to steric effects and hydroquinone monomers with large alkoxy substituents are not suited for the preparation of pillar[5]arenes. To solve this limitation, the most efficient approach is based on the post-functionalization of pillar[5]arene building block prepared in high yields from simple monomers. For this purpose, copper catalyzed alkyne-azide cycloaddition is particularly interesting [57]. This click reaction is effectively high yielding which is essential to achieve the efficient grafting of ten peripheral groups onto a single molecular scaffold [58]. Moreover, this chemistry is compatible with many functional groups thus allowing the grafting of a large variety of molecules. As typical examples, clickable pillar[5]arene scaffolds are depicted in Figure 2. These building blocks have been intensively used to prepare bioactive compounds and advanced materials [59,60,61,62,63,64,65]. They have been also functionalized with small dendrons to generate dendrimer-like nanostructures. These results are summarized in the next section.

## 3. Bioactive Dendrimers with a Pillar[5]Arene Core

### 3.1. Dendritic Gene Delivery Vectors with a Pillar[5]Arene Core

**Figure 3:**
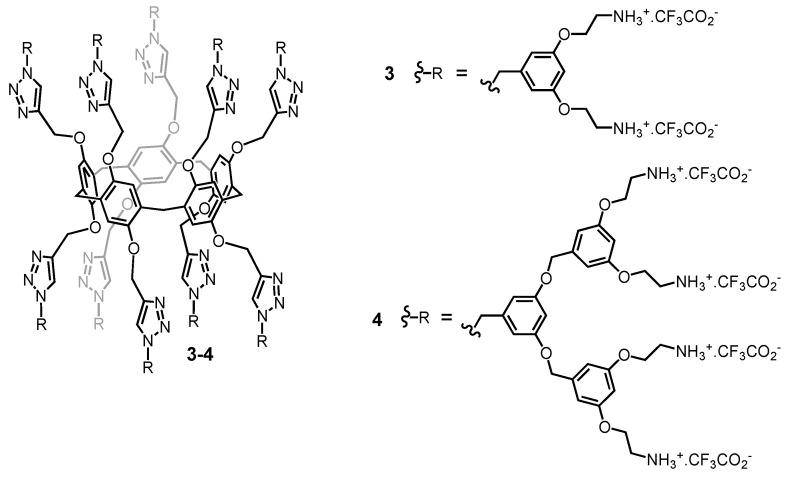
Dendronized pillar[5]arenes with peripheral ammonium functions (molecular weights (MW): 5787.41 (**3**); 11368.67 (**4**)). These polycationic derivatives have been used as non-viral gene vectors.

The first example of dendrimers with a pillar[5]arene core have been reported by our group [66]. Dendrons with peripheral Boc-protected amine functions have been grafted onto both rims of pillar[5]arene building block **1**. Subsequent treatment with trifluoroacetic acid (TFA) gave deprotected dendrimers **3** and **4** (Figure 3).

**Figure 4:**
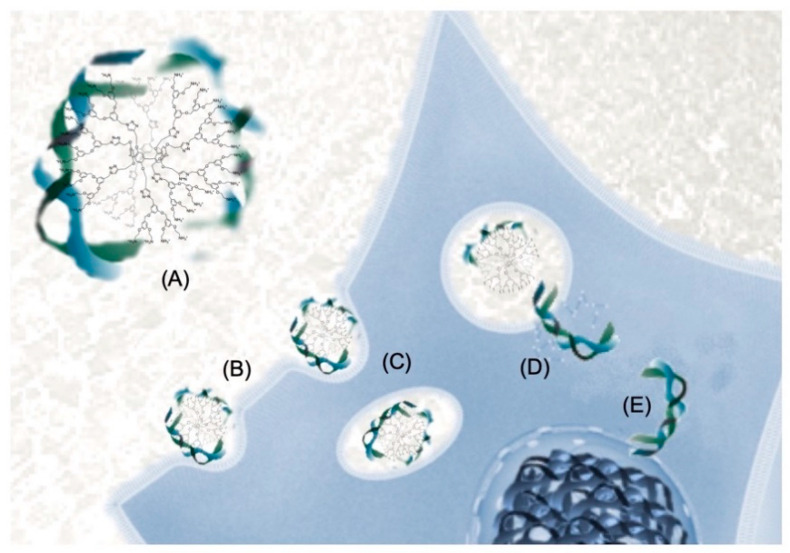
Schematic representation of the main step of gene delivery into a cell: (**A**) complexation of DNA; (**B**) interaction with the cellular membrane; (**C**) entry into the cell; (**D**) release into the cytosol; (**E**) intracellular transport into the nucleus where the DNA is expressed.

Dynamic light scattering (DLS) measurements revealed that both **3** and **4** form aggregates in water at concentrations higher than 3 nM. At concentrations lower than 1.5 nM, second-generation compound **4** no longer aggregates, whereas the first-generation analogue still forms nanoparticles with an average size of ca. 70 nm. Under these conditions, the hydrophobic interactions between the internal part of the dendrimers are vanished in the case of **4**, thus suggesting that the compound may adopt a nearly globular conformation despite the low generation number of the dendrons grafted onto the pillar[5]arene core. The ability of **3** and **4** to bind DNA has been evidenced by electrophoresis, DLS measurements, and transmission electron microscopy (TEM) [66,67,68]. Polyplexes have been prepared from plasmid DNA (pCMV-Luc) and **3** or **4**. Transfection efficiencies of the resulting self-assembled nanoparticles have been evaluated in vitro with HeLa cells (Figure 4). Practically useful levels of transfection have been obtained for both **3** and **4**. At the same time, the high levels of total cellular proteins observed in these experiments revealed very low toxicity. Interestingly, the first-generation dendrimer already has optimum gene delivery capabilities. In this particular case, high efficiency is not associated to high generation numbers to ensure DNA compaction into stable polyplexes suited for transfection experiments as typically observed for dendrimers [69]. This has been explained by the bolaamphiphilic character of **3** allowing to increase the stability of the nanoparticles formed with DNA by the establishment of additional hydrophobic interactions.

### 3.2. Glycoclusters Constructed on a Pillar[5]Arene Scaffold

**Figure 5:**
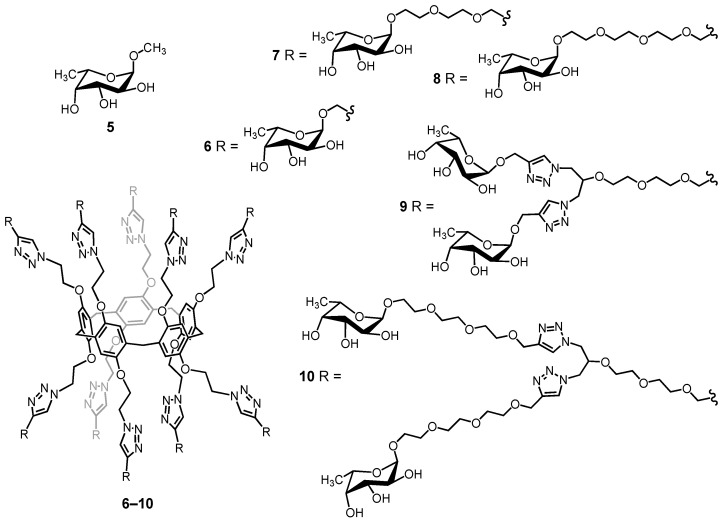
Model monovalent ligand **5** and glycopillar[5]arenes with 10 and 20 peripheral fucose subunits (MW: 3323.31 (**6**); 4204.41 (**7**); 4644.94 (**8**); 8028.18 (**9**); 10671.86 (**10**)).

**Figure 6:**
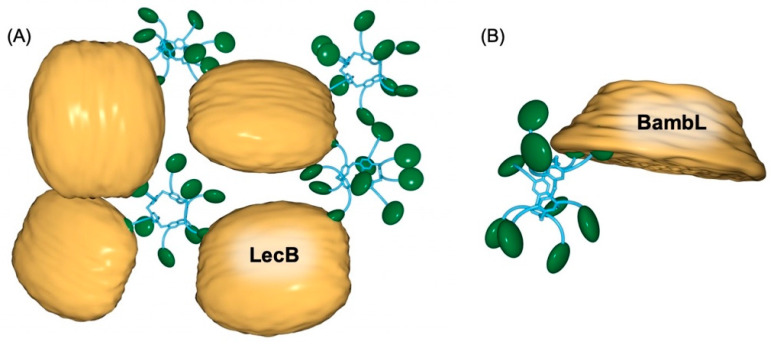
(**A**) Schematic representation of the clustering resulting from the association of a decavalent glycopillar[5]arene derivative with Lec A. (**B**) Schematic representation of the simultaneous binding of two fucose units of a decavalent glycopillar[5]arene derivative to BambL.

Pillar[5]arene scaffolds have been also used to prepare glycoclusters decorated with ten peripheral sugar residues [70,71,72,73,74]. These compounds have been assayed as multivalent ligands for various bacterial lectins and large binding enhancements have been evidenced through the well-established glycoside cluster effect [75,76,77,78,79]. To further increase the valency of the glycopillar[5]arene derivatives and hopefully their binding capabilities, first-generation glycodendrons have been grafted onto the pillar[5]arene core. A series of fucosylated compounds have been prepared from building block **2** and first-generation dendrons with an alkyne function at the focal point. Glycoclusters **9** and **10** with 20 peripheral fucose subunits are depicted in Figure 5 together with their related decavalent systems **6**–**8** and model compound **5**. Compounds **5**–**10** have been assayed towards two fucolectins, namely LecB from *Pseudomonas aeruginosa* and BambL from *Burkholderia ambifaria*. These studies have been carried out by A. Imberty and co-workers [74]. The dissociation constants (*K*_D_) derived from isothermal titration microcalorimetry (ITC) experiments are summarized in Table 1. LecB is typically not very sensitive to the multivalent presentation of fucose residues [74]. This is explained by the topology of this lectin. The four binding pockets are actually quite far from each other, thus preventing the simultaneous binding of two fucose subunits of a multivalent ligand. As a result, binding enhancement resulting from chelate cooperativity is not possible in this particular case. As anticipated, there is no dramatic improvement when going from monovalent ligand **5** to the multivalent fucosylated pillar[5]arenes **6**–**10**. Decavalent compound **6** is even a weaker ligand, most probably because of steric effects resulting from the short spacer that prevents optimal interactions of the fucose residue in the binding pocket of LecB. The small binding enhancement observed for **7**–**10** results exclusively from aggregation, as shown in Figure 6.

In this case, simultaneous binding of two fucose moieties of one glycopillar[5]arene occurs to two different LecB proteins within the same aggregate. This is beneficial from an enthalpic point of view. However, the positive enthalpic effect is largely counterbalanced by a strong entropic penalty. As a result, the overall enhancement is rather weak when going from **5** to **6**–**10**.

The topology of BambL is very different. In this case, six binding pockets are located on the same face of the protein and the observed binding enhancement resulting from the multivalent presentation of fucose residues with **6–10** when compared to monovalent model **5** is likely mainly due to chelate cooperativity (Figure 6). The beneficial enthalpic contribution resulting from chelate cooperativity is, however, affected by an entropic penalty due to clustering. This is particularly true for ligands **9** and **10** with 20 peripheral fucose residues. Overall, the valency number plays an important role in the affinity of multivalent ligands **6–10** towards LecB and BambL, but the nature of the linker unit between the core and the peripheral fucose moieties is also important. Nonetheless, compounds **6–10** are amongst the most potent ligands for LecB and BambL reported to date, thus showing the potential of glycopillar[5]arenes for therapeutic applications based on an anti-adhesive strategy [75,76,77,78,79].

## 4. Dendritic Liquid-Crystalline Pillar[5]Arenes

**Figure 7:**
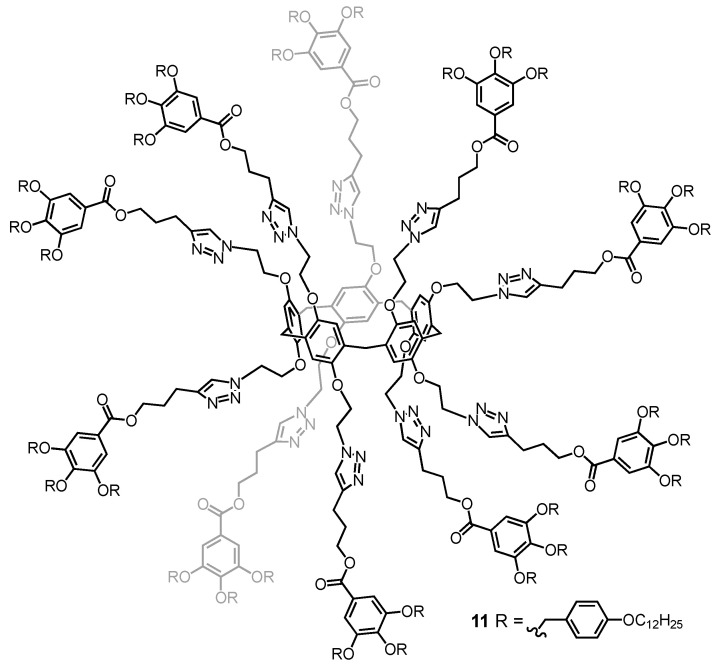
Dendronized pillar[5]arene **11** (MW: 11896.72).

**Figure 8:**
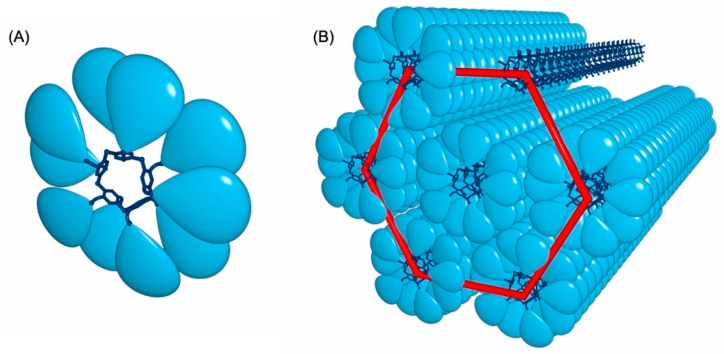
(**A**) Schematic representation of compound **11**. (**B**) Supramolecular arrangement of compound **11** into the hexagonal columnar phase, the dendrons have been omitted in one column (top right) to highlight the nanotubular arrangement of the pillar[5]arene cores.

Pillar[5]arenes are attractive five-fold symmetrical hard-core units for the construction of original liquid-crystalline materials. The first examples have been constructed by grafting cyanobiphenyl-based mesogenic units onto the pillar[5]arene core [82,83]. The same design principle has been also used to design switchable liquid-crystalline derivatives by incorporating azobenzene-containing mesogenic moieties on both rims of the pillar[5]arene core [84]. In all the cases, smectic organization have been observed in the liquid-crystalline phase. In contrast, when the pillar[5]arene core has been functionalized with Percec-type poly(benzylether) dendrons [85], the peripheral subunits promote a totally different supramolecular organization [86,87]. A typical example of dendronized pillar[5]arene derivative is depicted in Figure 7. X-ray diffraction measurements revealed a supramolecular organization into a hexagonal columnar liquid-crystalline mesophase. The chemical information stored in the peripheral dendrons of **11** actually drives the conformational equilibrium towards the formation of a disc-like tertiary structure. As a result, the compound adopts a perfect shape, allowing the self-organization into columnar assemblies in which one molecule forms an entire disc, as schematically shown in Figure 8. Interestingly, the columnar assembly generates infinite self-assembled nanotubes despite the generated free volume. This work represents, therefore, a first step towards the preparation of a new class of organic nanotubes.

## 5. Self-Assembled Dendrimers

**Figure 9:**
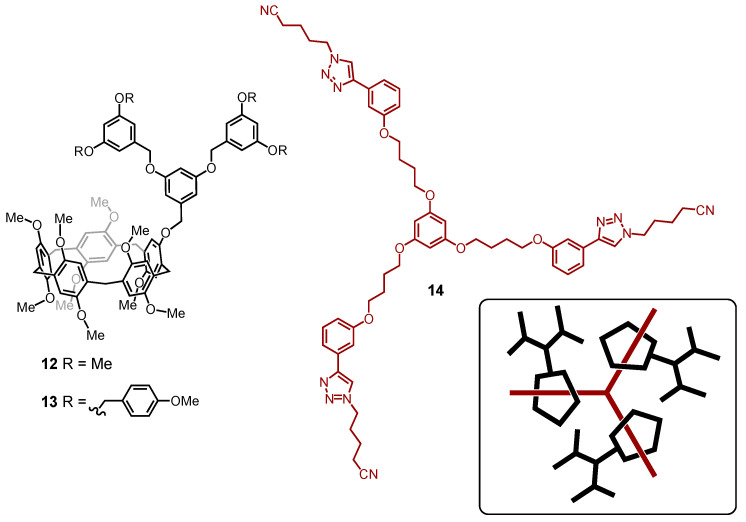
Dendronized pillar[5]arene derivatives **12** and **13**, and tritopic ligand **14**. The inset shows a schematic representation of the self-assembled dendrimer resulting from the binding of **14** with three pillar[5]arenes **13** (MW: 1159.34 (**12**); 1463.73 (**13**)).

With their tubular structures, pillar[5]arene macrocycles are well-suited hosts for the formation of inclusion complexes with a large variety of guests [88]. Jia and Li used the complexation ability of pillar[5]arenes to self-assemble supramolecular dendrimers [89]. First and second-generation dendrons have been grafted onto a monohydroxylated pillar[5]arene derivative to generate compounds **12** and **13**, respectively (Figure 9). Their self-assembly with a tritopic connector (**14**) has been investigated in CDCl_3_ solutions by ^1^H NMR binding studies. The 3:1 host-guest assemblies are rather stable under these conditions. The formation of the star-shaped dendritic trimers has been further confirmed by their diffusion coefficients estimated by diffusion-ordered NMR spectroscopy (DOSY).

Finally, one should also mention the outstanding work of Yang and co-workers on organometallic rotaxane dendrimers constructed with mechanically interlocked pillar[5]arene moieties [90,91,92,93,94,95,96]. These compounds are out of the scope of the present paper and this chemistry has been summarized in recent review articles [97].

## 6. Conclusions

Clickable pillar[5]arenes are versatile building blocks for the preparation of multifunctional nanomaterials. Whereas only a very few examples of dendronized derivatives have been reported so far, the easy access to dendritic-like structures with limited synthetic efforts is very attractive for future applications. Pillar[5]arene-containing dendrimers have already been used to generate non-viral gene delivery systems, bioactive glycoclusters, and liquid-crystalline materials. These results pave the way to new generations of more sophisticated advanced materials and bioactive compounds. Examples include heteroglycoclusters targeting several proteins and new gene vectors with additional functions such as sugars for cell targeting or fluorescent moieties for monitoring cell uptake. The host-guest properties of the pillar[5]arene macrocycle also open additional perspectives for the design of new supramolecular functional dendrimers for different applications.

## Figures and Tables

**Table 1:** Isothermal titration microcalorimetry data for the interactions of glycopillar[5]arenes with LecB and RSL.

Ligand	Valency	*K*_D_ (nM)	β ^1^
LecB from *Pseudomonas aeruginosa* ^2^			
**5** ^3^	1	430	1
**6**	10	990	0.4
**7**	10	220	1.9
**8**	10	280	1.5
**9**	20	150	2.9
**10**	20	180	2.4
BambL from *Burkholderia ambifaria* ^2^			
**5** ^4^	1	960	1
**6**	10	60	16
**7**	10	19	50
**8**	10	57	17
**9**	20	17	56
**10**	20	27	36

^1^ Calculated as the ratio of the value obtained for monovalent reference compound **5** to the value of the ligand. ^2^ From [74]. ^3^ From [80]. ^4^ From [81].

## Data Availability

Not applicable to this article.

## Sample Availability

Not applicable to this article.
